# The Use of 3D-Printed Custom-Made Implants as an Attractive Potential Alternative to the Treatment of Segmental Bone Loss in Foot and Ankle

**DOI:** 10.3390/jcm11164738

**Published:** 2022-08-13

**Authors:** Silvio Caravelli, Giuseppe Ambrosino, Alberto Grassi, Stefano Zaffagnini, Massimiliano Mosca

**Affiliations:** II Clinic of Orthopaedics and Traumatology, IRCCS Istituto Ortopedico Rizzoli, 40136 Bologna, Italy

## 1. Introduction

The treatment of segmental tibial and ankle bone loss after radical surgery for chronic osteomyelitis is one of the most challenging problems encountered by orthopaedic surgeons. Open tibia and ankle fractures occur with an incidence of 3.4 per 100,000 and 1.6 per 100,000, respectively [1,2] and have a high propensity to lead to the development of fracture-related infection with associated chronic osteomyelitis. 

Once infection is established, there is often associated fracture non-union, limb length discrepancy, and deformity with the early development of osteoarthritis. This completely changes the prognosis and determines the delay in the clinical recovery of the patient.

In cases where there is significant contamination or bacterial load, infection with more virulent or multiple organisms, or complicating patient comorbidities, a two-stage reconstruction may be the best treatment option. The first approach includes the Masquelet technique, Papineau technique, or antibiotic-impregnated internal fixation techniques [3]. 

When the infection has been cleared and the wound bed is healthy, reconstruction includes multiple options: autograft bone transport utilizing external fixation [4], vascularized fibular transfer [5], massive cancellous bone grafting with and without tissue transfer [6], and osteomyocutaneous flaps.

In this surgical arena, we introduce a discussion regarding 3D-printed technology with custom-made spacer implants. 

## 2. Discussion

Given the complexity and uniqueness of each individual case, multiple factors, including patient comorbidities, injury type, soft tissue or bone defects, as well as the chronicity of the infection, must be considered. Because of these factors, there is no single solution for any case. Individualized treatment regimens must be considered. 

Combining 3D-printed technology and the experience of the surgeon allows the development of custom-made implants that, when positioned into the bone loss space to ensure a proper fit, are able to replace the resected bone and restore the proper alignment and length of the lower limb.

The production of each implant starts with the measurement of the contralateral tibia and ankle radiographs, MRI, and/or computed tomography (CT) scans. The CT images are then collected to create a computerized 3D model, the implant’s first prototype. 

The custom-made spacer consists of a trabecular metal tibial component, an implant for the reconstruction of the previously resected portion of bone, and eventually of talus if the intra-articular defect exists, associated to tibial and talar cutting guides to prepare regular resections, and to a drilling guide for the screws used to fix the implant. The whole system is then stabilized through a tibiotalocalcaneal retrograde nail. These custom-made spacer implants require up to 4 to 6 weeks to be produced.

The type of surgical approach is important to reduce rehabilitation morbidity for the patient. The technical difficulties, the particular geometry of the resected bone, and the altered anatomy of the soft tissues can influence the surgical result. Despite the design being developed to use the residual bone as a reference for the level of the cut and the patient-specific guide for the bone as a rotation reference, minimal changes during the surgery can create various problems when fitting the implant. However, compared to the more commonly used technique of bone transport with external fixation, no complications (including non-union, stress, or refracture at the bone regeneration site or pin tract infections) have been observed. An extended non-weight-bearing period is also not needed [7]. Using an autograft has the considerable disadvantages of donor site morbidity and limited quantity [8,9]; moreover, the allograft could be potentially limited in size and less osteogenic, with higher rates of non-union [10]. Both have been known for the possibility of undergoing late collapse and can be limited by the difficulty in achieving the correct shape for reconstruction [11,12].

The economic aspect is a limiting factor regarding the spread of patient-specific implants. Custom-made systems by definition are not mass-produced and require a multidisciplinary team that deals with the design and production of the implants.

In conclusion, post-traumatic infections pose a daily challenge for orthopaedic surgeons and patients alike. A multidisciplinary approach is required with high levels of coordination between specialties. The customizability of 3D printing is an attractive potential alternative to the treatment of segmental bone loss in the foot and ankle. The use of 3D-printed implants can avoid the complications and limitations brought about by autografts and allografts in foot and ankle surgery. Furthermore, value-driven models of care may favour the adoption of 3D technology as it becomes more accessible. Long-term studies are needed to monitor delayed complications such as stress shielding and implant failure.

## References

[1] Court-Brown C.M., Bugler K.E., Clement N.D., Duckworth A.D., McQueen M.M. (2012). The epidemiology of open fractures in adults. A 15-year review. Injury.

[2] Elniel A.R., Giannoudis P.V. (2018). Open fractures of the lower extremity: Current management and clinical outcomes. EFORT Open Rev..

[3] Jonard B., Dean E. (2017). Posttraumatic Reconstruction of the Foot and Ankle in the Face of Active Infection. Orthop. Clin. N. Am..

[4] Naggar L., Chevalley F., Blanc C.H., Livio J.J. (1993). Treatment of large bone defects with the Ilizarov technique. J. Trauma.

[5] Chung Y.K., Chung S. (1998). Ipsilateral island fibula transfer for segmental tibial defects: Antegrade and retrograde fashion. Plast. Reconstr. Surg..

[6] Cierny G., Zorn K.E. (1994). Segmental tibial defects. Comparing conventional and Ilizarov methodologies. Clin. Orthop. Relat. Res..

[7] Uzel A.P., Lemonne F., Casoli V. (2010). Tibial segmental bone defect reconstruction by Ilizarov type bone transport in an induced membrane. Orthop. Traumatol. Surg. Res..

[8] Boone D.W. (2003). Complications of iliac crest graft and bone grafting alternatives in foot and ankle surgery. Foot Ankle Clin..

[9] Masquelet A.C., Fitoussi F., Begue T., Muller G.P. (2000). Reconstruction of the long bones by the induced membrane and spongy autograft. Ann. Chir. Plast. Esthet..

[10] McGarvey W.C., Braly W.G. (1996). Bone graft in hindfoot arthrodesis: Allograft vs autograft. Orthopedics.

[11] Bouchard M., Barker L.G., Claridge R.J. (2004). Technique tip: Tantalum: A structural bone graft option for foot and ankle surgery. Foot Ankle Int..

[12] Conti S.F., Wong Y.S. (2002). Osteolysis of structural autograft after calcaneocuboid distraction arthrodesis for stage II posterior tibial tendon dysfunction. Foot Ankle Int..

